# Comparative virulome analysis of four Staphylococcus epidermidis strains from human skin and platelet concentrates using whole genome sequencing

**DOI:** 10.1099/acmi.0.000780.v3

**Published:** 2024-04-03

**Authors:** Basit Yousuf, Annika Flint, Kelly Weedmark, Franco Pagotto, Sandra Ramirez-Arcos

**Affiliations:** 1 Medical Affairs and Innovation, Canadian Blood Services, Ottawa, Canada; 2 Department of Biochemistry, Microbiology and Immunology, University of Ottawa, Ottawa, Canada; 3 Bureau of Microbial Hazards, Health Products and Food Branch, Health Canada, Ottawa, Canada

**Keywords:** biofilm, platelet concentrates, phylogeny, *Staphylococcus epidermidis*, virulome, whole genome sequencing

## Abstract

*Staphylococcus epidermidis* is one of the predominant bacterial contaminants in platelet concentrates (PCs), a blood component used to treat bleeding disorders. PCs are a unique niche that triggers biofilm formation, the main pathomechanism of *S. epidermidis* infections. We performed whole genome sequencing of four *S. epidermidis* strains isolated from skin of healthy human volunteers (AZ22 and AZ39) and contaminated PCs (ST10002 and ST11003) to unravel phylogenetic relationships and decipher virulence mechanisms compared to 24 complete *S. epidermidis* genomes in GenBank. AZ39 and ST11003 formed a separate unique lineage with strains 14.1 .R1 and SE95, while AZ22 formed a cluster with 1457 and ST10002 closely grouped with FDAAGOS_161. The four isolates were assigned to sequence types ST1175, ST1174, ST73 and ST16, respectively. All four genomes exhibited biofilm-associated genes *ebh*, *ebp*, *sdrG*, *sdrH* and *atl*. Additionally, AZ22 had *sdrF* and *aap*, whereas ST10002 had *aap* and *icaABCDR*. Notably, AZ39 possesses truncated *ebh* and *sdrG* and harbours a toxin-encoding gene. All isolates carry multiple antibiotic resistance genes conferring resistance to fosfomycin (*fosB*), β-lactams (*blaZ*) and fluoroquinolones (*norA*). This study reveales a unique lineage for *S. epidermidis* and provides insight into the genetic basis of virulence and antibiotic resistance in transfusion-associated *S. epidermidis* strains.

## Graphical Abstract

Virulome profile of four *Staphylococcus epidermidis* strains isolated from platelet concentrates and human skin.

## Data Summary

The complete *de novo* genome sequences of the four *Staphylococcus epidermidis* strains used in this study have been uploaded to GenBank under accession numbers CP071988.1, CP071992.1, CP071994.1 and CP071996.1 corresponding to strains ST10002, ST11003, AZ22 and AZ39, respectively. All genome sequences are available in GenBank under BioProject ID PRJNA703008.

**Impact Statement**

Platelet concentrate (PC) transfusions play a crucial role in the management of haematological disorders and cancers, yet their significance is compromised by the persistent threat of bacterial contamination, occasionally leading to post-transfusion septic reactions. This study investigates the virulome profiles of four *Staphylococcus epidermidis* strains originating from human skin and PCs, employing comparative genomics to explore their genomic disparities. The study focuses on *S. epidermidis* strains AZ22, AZ39, ST10002 and ST11003, two isolated from skin and two from PCs. Among these, AZ22, AZ39 and ST10002 exhibit biofilm positivity in trypticase soy broth (TSB) supplemented with glucose (TSBg), while ST11003 is a biofilm-negative strain. Interestingly, all strains display enhanced biofilm formation in PCs, underscoring the need to understand their genomic distinctions for unravelling their impacts on pathogenicity. Genomic analysis classifies these strains into sequence types ST73, ST1175, ST16 and ST1174, respectively, according to the PubMLST database. Phylogenetic analysis reveals a distinct clade formed by ST11003 and AZ39, while ST10002 and AZ22 form separate clusters. Furthermore, the study elucidates the genetic basis of virulence and antibiotic resistance in transfusion-associated *S. epidermidis* strains. These strains offer valuable insights as model strains for subsequent *in vitro* and *in vivo* studies examining the physiology of *S. epidermidis*.

## Introduction

*Staphylococcus epidermidis* is a coagulase-negative staphylococcal species (CoNS) that represents a predominant constituent of the human skin microbiota [1]. It is an important opportunistic pathogen in healthcare settings and causes bloodstream- and medical device-related infections (National Nosocomial Infections Surveillance System, 2004)[2]. *S. epidermidis* has also been reported to be the predominant aerobic contaminant in platelet concentrates (PCs), a blood component used to treat thrombocytopenic patients. Importantly, *S. epidermidis* has been involved in fatal transfusion reactions [3,5]. * S. epidermidis* is introduced from skin into the donated blood during venipuncture and reaches clinically significant levels (i.e. > 10^5^ c.f.u. ml^–1^) during standard PC storage at temperatures of 20–24 °C, under agitation for up to 7 days in gas-permeable plastic containers [6,7].

The main pathomechanism of *S. epidermidis* infections is the formation of surface-attached aggregates known as biofilms [8]. Importantly, it has been demonstrated that the PC storage environment can promote biofilm formation by *S. epidermidis* isolates considered to be biofilm-negative in lab-based studies [9]. Biofilms are formed in PCs by adhering to the inner surface of PC storage containers or by a direct interaction of bacterial cells with activated platelets [10].

Numerous *S. epidermidis* strains produce a biofilm matrix mainly composed of polysaccharide intercellular adhesin (PIA), which is synthesized by enzymes encoded by the *icaADBC* operon, under the control of its cognate repressor protein IcaR [11]. Some strains produce PIA-independent biofilms with a matrix mainly composed of proteins while some isolates produce biofilms with matrices composed of both polysaccharides and PIA. Biofilm-associated proteins include the accumulation-associated protein (Aap) and the extracellular matrix binding protein (Embp) [12,13]. Biofilm matrices composed of PIA, Aap or Embp vary significantly in their morphology and thus could respond differently to immune challenges [14]. Additionally, extracellular DNA (eDNA) also forms part of the staphylococcal biofilm matrix [15]. Strains which harbour both *ica* and *aap* genes form stronger biofilms in comparison to strains possessing only one of them [16].

*S. epidermidis* biofilms formed during PC storage have increased virulence as demonstrated in a nematode killing assay. Also, *S. epidermidis* biofilms formed in PCs are resistant to the bactericidal action of platelet-derived antimicrobial peptides (AMPs) and display structural changes of the cell wall and biofilm matrix [17,20]. Recently, we have demonstrated upregulation of *S. epidermidis* antibiotic resistance genes induced by the PC storage environment which may challenge treatment of infected transfusion patients [20]. Antibiotic-resistant *S. epidermidis* strains from diverse geographical habitats of different countries mostly belong to the sequence types (ST) ST2, ST5, ST12, ST23 and ST59 [21,22]. The antibiotic resistance and virulence genes in *S. epidermidis* are acquired and transmitted through mobile genetic elements (MGEs) such as plasmids, transposons, insertion sequences (ISs), phages and pathogenicity islands [23,24]. The pathogenicity island carrying the arginine catabolic mobile element (ACME) is transmitted through horizontal gene transfer from *S. epidermidis* to *Staphylococcus aureus* [25,26]. ACME plays a pivotal role in facilitating host colonization and immune evasion.

Despite the prevalence of *S. epidermidis* as a predominant bacterial contaminant in PCs, its phylogeny and epidemiology with respect to PCs remains unknown. Advancing knowledge in the ecology of *S. epidermidis* in the unique PC milieu will help to understand growth and biofilm formation of this bacterium with implications for virulence for transfusion patients. In this study, we performed whole genome sequencing (WGS) of four *S. epidermidis* strains, including two isolated from the skin of healthy human volunteers and two from contaminated PCs, to gain insight into their phylogeny and virulence mechanisms.

## Methods

### Bacterial strains and growth conditions

Four *S. epidermidis* isolates from the Canadian Blood Services strain collection were studied including two isolates from the skin of healthy volunteers, which had not been in contact with blood components (AZ22, AZ39) and two from contaminated PCs (ST10002, ST11003). The biofilm formation ability of these strains has been previously characterized [27]. Strains AZ22, AZ39 and ST10002 produce biofilms in trypticase soy broth (TSB) supplemented with glucose (TSBg), while all four strains produce biofilms in PCs [27]. *S. epidermidis* AZ22, AZ39 and ST11003 are PIA negative while strain ST10002 is PIA positive; biochemical assays demonstrated that PIA-negative strains formed biofilms with matrices mainly composed of proteins and eDNA [19].

### DNA extraction and whole genome sequencing

Single colonies of *S. epidermidis* strains were picked up from trypticase soy agar (TSA) plates, inoculated in 5 ml TSB with 0.6 % yeast extract and incubated at 35 °C overnight. DNA was extracted using the Zymo Quick-DNA HMW MagBead kit (Cedarlane) with lysozyme and RNase A treatment according to the manufacturer’s instructions (Zymo Research). DNA was quantified using a Qubit fluorometer (Fisher).

*S. epidermidis* Illumina libraries were constructed using the NexteraXT DNA Library Preparation Kit and Nextera XT Index Kit v2 (Illumina). Paired-end Illumina sequencing was performed on a MiSeq instrument (v3 chemistry, 2×300 bp) (Illumina). * S. epidermidis* Nanopore libraries were constructed using the Rapid Barcoding Sequencing kit (SQK-RBK004; Oxford Nanopore Technologies). Libraries were sequenced on a 1D MinION device (R9.4, FLO-MIN106) using a FLO-MIN106 flow cell for 16 h. Signal processing, base calling, demultiplexing and adapter trimming were performed using Guppy (Guppy GPU v3.3.3+fa743 ab). Whole genome *de novo* assembly of *S. epidermidis* AZ22, AZ39, ST10002 and ST11003 isolates was performed at the Bureau of Microbial Hazards, Health Canada (Ottawa, ON, Canada).

### Bioinformatic analyses

Illumina reads were processed using Fastp (v0.20.0) [28] to remove adapter and barcode sequences, correct mismatched bases in overlaps, and filter low-quality reads. Nanopore reads were processed using Filtlong (v0.2.0, github.com/rrwick/Filtlong) to remove reads <1 kb. Hybrid assemblies using the Illumina and Nanopore filtered reads were performed using Unicycler (v0.4.8) [29] in normal mode for strains ST10002, ST11003 and AZ22 or Trycycler (v0.3.3, github.com/rrwick/Trycycler/wiki) for strain AZ39. Briefly for Trycycler, Flye (v2.8.1-b1676) [30], Minipolish+Miniasm (v0.1.2) [31], Wtdbg2 (v2.5) [32] and Raven (v1.2.2, github.com/lbcb-sci/raven) were used to construct long-read assemblies. A consensus genome was produced from the long-read assemblies using the cluster, reconcile, partition and consensus functions of Trycycler. Error correction of the assembled genomes was performed using Medaka (v1.1.3, github.com/nanoporetech/medaka) and the Nanopore long reads followed by Pilon (v1.23) [33] and Illumina short reads. Assembled genomes were annotated using PGAP (2020-09-24.build4894; best-placed reference protein set; GeneMarkS-2+; github.com/ncbi/pgap) and analysed using Quast (v5.0.2, github.com/ablab/quast).

### Phylogenetic analysis

Phylogenetic analysis was performed for the four *S. epidermidis* assembled genomes against the 24 fully assembled genomes of *S. epidermidis* available on GenBank (accessed in January, 2022). Core genomes of all these strains were aligned using Parsnp (v1.2) [34]. Maximum likelihood phylogenetic trees based on the Tamura–Nei model were reconstructed and visualized with mega (v11.0.10) [35] using the aligned sequences and 1000 bootstrap replicates.

### *Insilico* analysis

The CRISPRfinder tool [36] was used to find clustered regularly interspaced short palindromic repeats (CRISPRs) in the four *S*. *epidermidis* strains. Pathogenicity of these strains towards the human host was predicted using PathogenFinder (http://www.genomicepidemiology.org/services/). Similarly, antimicrobial resistance genes were identified using ResFinder 4.1 (minimum length and threshold of 60 and 90 %, respectively), and the Comprehensive Antibiotic Resistance Database (CARD) [37]. Prophages were identified using PHASTER analysis [38]. Virulence genes present in these strains were analysed using the VF-DB database (http://www.mgc.ac.cn/VFs/) [39]. We used PGAP and the nocoRNAc program to predict non-coding RNAs (ncRNAs), based on cmsearch (v1.1.1) [40,41].

### Multilocus sequence typing

Four *S. epidermidis* strains were checked for multilocus sequence typing (MLST) using the PubMLST database [42]. Assignments to STs was based on matching with the internal fragments of seven housekeeping genes (*arcC, aroE, gtr, mutS, pyrR, tpiA* and *yqiL*) [43].

## Results

### Genome characteristics

Characteristics of the four *S. epidermidis* genomes, which include size of chromosome and plasmids, GC content, predicted protein coding genes, and number of rRNAs and tRNAs, are listed in Table 1. The size of the genomes ranged from 2.4 to 2.5 Mb with a GC content of 32 %, comparable to other known *S. epidermidis* strains. The GC skew of the AZ22, ST10002 and ST11003 genomes is asymmetrical and similar to the genomes of *S. epidermidis* RP62A and O47 [44,45] (Fig. S1, available in the online version of this article).

**Table 1.** Overall genome structure of the four *S. epidermidis* strains isolated from human skin and PCs

Isolate	Chromosome size(bp)	Plasmid size(bp)	rRNAs	No. of CDS		tRNAs	Pseudogenes	%GC	Source
				Total Hypothetical					
ST10002	2 439 283	20813, 9891, 3265	19	2399	237	61	66	32.19	PCs
ST11003	2 566 999	24 268	19	2261	201	60	62	32.17	PCs
AZ22	2 462 009	13 277	19	2284	203	61	70	32.23	Skin
AZ39	2 454 327	41582, 8761, 2321	19	2314	213	60	184	32.13	Skin

### Multilocus sequence typing

*S. epidermidis* AZ22, AZ39, ST10002 and ST11003 genomes were assigned to ST73, ST1175, ST16 and ST1174, respectively, based on the PubMLST database. The STs of 24 reference genomes along with the collection site source are detailed in Table S1.

### Non-coding RNAs

The numbers of ncRNAs identified with nocoRNAc were higher than predicted by the PGAP program due to the use of the more stringent Rfam database (Table S2). All four strains showed the presence of RNAIII, RsaA, RsaD, RsaE and RsaH. Overexpression of RsaE has been reported to promote PIA-mediated biofilm formation as well as release of eDNA [46].

### Identification of pathogenicity, CRISPRs and MGEs

All four *S. epidermidis* strains were predicted to be pathogenic based on PathogenFinder with mean probabilities of 0.949, 0.957, 0.954 and 0.956 for ST10002, ST11003, AZ22 and AZ39, respectively.

CRISPRs have a role in preventing plasmid transformation and conjugation [47]. *S. epidermidis* ST10002 possesses four CRISPRs and four spacers, ST11003 has three CRISPRs and three spacers, AZ22 has two CRISPRs and two spacers, and AZ39 carries one CRISPR and one spacer (Table 2). Notably, CRISPR-associated (*cas*) genes (*cas1, cas2* and *cas6*) were not observed in any of these genomes. CRISPR genes are the main barriers to horizontal gene transfer and protect bacteria from phage infections [47]. No restriction modification systems were identified in any of the strains.

**Table 2.** MLST and genotypic characteristics of the four *S. epidermidis* strains

Isolate	Sequence type (MLST)	Resistome	Plasmid replicon type	RM*	**Mobile genetic elements**	CRISPR†	No. of codons with frameshift
ST10002	16	*norA*, *fosB msr*(A), *aph*(3′)-III, *mph*(C), *blaZ*, *qacA*, *SAT*-4	rep39	–	ACME	4	42
ST11003	1174	*norA*, *fosB msr*(A), *blaZ*, *qacB*	rep39	–	ACME	3	40
AZ22	73	*norA*, *fosB*, *dfrC*	Unknown	–	ACME	1	40
AZ39	1175	*norA*, *fosB*	Unknown	–	ACME	2	150

* RM- R, restriction modification.

† CRISPR-, Type III-A CRISPR-Cas system.

The genomes of *S. epidermidis* ST10002, ST11003 and AZ39 contain two prophages whereas AZ22 has three prophages (Table 3, Fig. S2). The ACME mobile element was present in all four genomes.

**Table 3.** Prophages of the four *S. epidermidis* isolates

Strain	Prophage	Size (kb)	Status
AZ22	PHAGE_Staphy_StB20_like_NC_028821	50.7	Complete
	PHAGE_Achrom_JWAlpha_NC_023556	5.8	Incomplete
	PHAGE_Bacill_SP_15_NC_031245	15.8	Incomplete
AZ39	PHAGE_Staphy_PT1028_NC_007045	22.8	Incomplete
	PHAGE_Staphy_CNPH82_NC_008722	20.3	Incomplete
ST10002	PHAGE_Strept_20617_NC_023503	5.8	Incomplete
	PHAGE_Staphy_PT1028_NC_007045	22	Questionable
ST10003	PHAGE_Staphy_187_NC_007047	43.6	Questionable
	PHAGE_Staphy_StB12_NC_020490	57.9	Intact

### Global regulatory systems

Different global regulatory systems pivotal in biofilm formation, cell wall biosynthesis and controlling expression of repertoire of virulence factors were observed in the four *S. epidermidis* genomes, as listed in Table 4. The accessory gene regulator (*agr*), the master regulator of *Staphylococcus* virulence, is encoded by the four-gene operon *agrABCD*, which is present in the ST11003, ST10002 and AZ22 strains, whereas *agrB* and *agrC* possess a frameshift mutation in *S. epidermidis* AZ39. Genes coding for the SaeRS two-component regulatory system, which negatively regulates biofilm formation, were only observed in *S. epidermidis* ST11003 (Table 4).

**Table 4.** One- and two-component global regulatory systems in the four *S. epidermidis* strains

Isolate	*saeRS*	*mgrA*	*sigB*	*vraSR*	*spxA*	*lytRS*	*luxS*	*graSRX*
ST11003	+	+	+	+	+	+	+	*+*
ST10002	−	+	+	+	+	+	+	*+*
AZ22	−	+	+	+	+	+	+	*+*
AZ39	−	+	+	+	+	+	+	*+*

### Molecular phylogeny and comparison of virulence factors amongst the strains

**Fig. 1.**
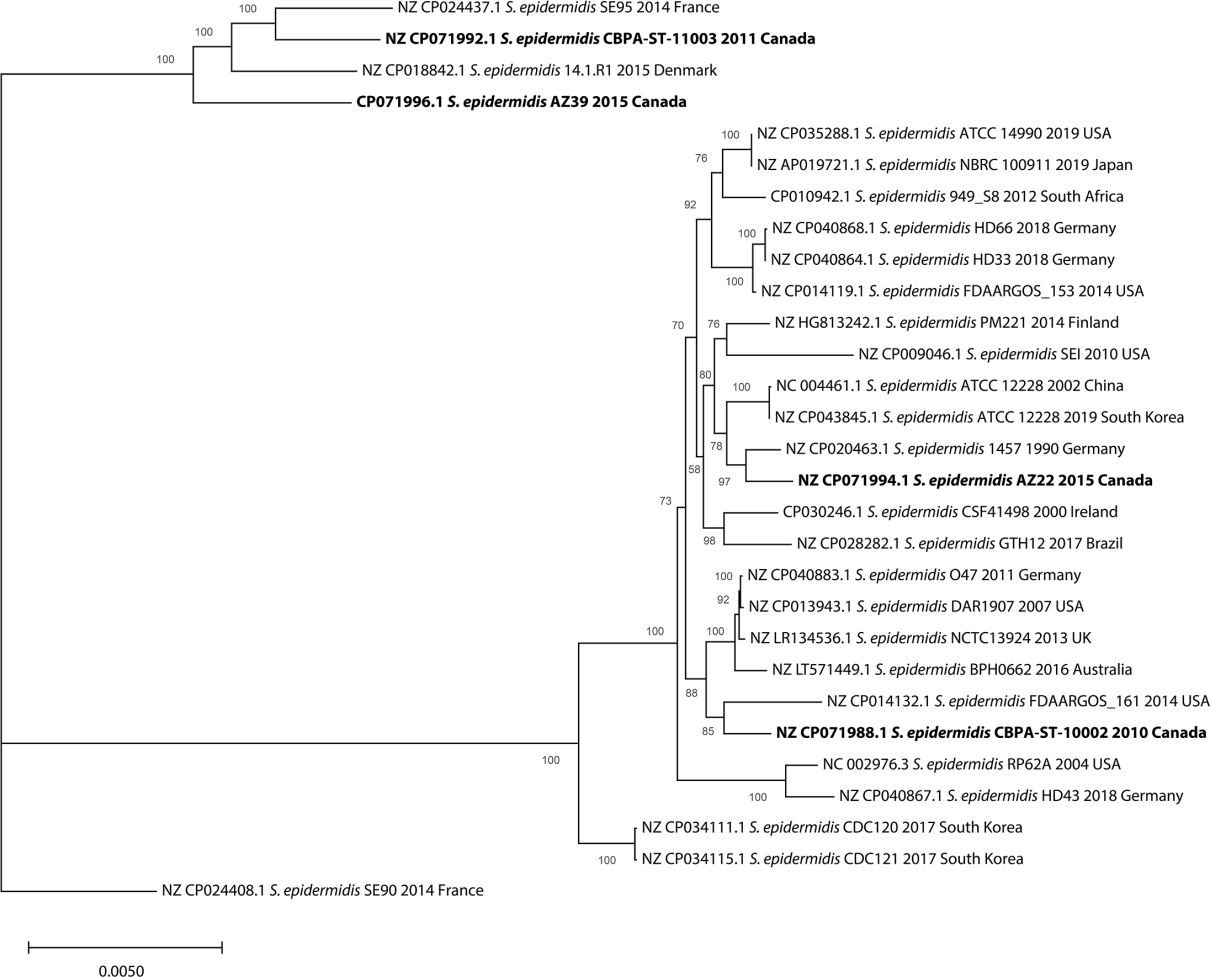
Molecular phylogeny based on *S. epidermidis* whole genome sequences. The core-genome multi-alignment was generated using Parsnp and maximum likelihood tree reconstructed using mega (v11.0.10) with 1000 bootstrap replicates. Bar, the phylogenetic distance expressed as nucleotide substitutions per site. Bootstrap values are shown at each node. Reference sequences were obtained from GenBank with accession numbers, strain, year and country of isolation shown. Canadian *S. epidermidis* strains are shown in bold.

The four isolates formed three separate phylogenetic clades. *S. epidermidis* ST10002 showed close relatedness to the FDAARGOS_161 strain, which was isolated from peripheral blood [48], and grouped in the cluster with *S. epidermidis* O47. Strain AZ22 formed a separate cluster with *S. epidermidis* 1457 and ATCC 12228 strains. Remarkably, ST11003 and AZ39 grouped in a separate unique phylogenetic clade with *S. epidermidis* SE95 and 14.1 .R1 reported from France and Germany (Fig. 1).

Virulence in *S. epidermidis* is mainly attributed to biofilm biosynthesis and its ability to evade the innate immune response. We compared the virulence factors amongst the members of the three separate phylogenetic clusters to identify signature factors of each cluster. For the initial biofilm attachment phase, *S. epidermidis* produces a repertoire of cell wall anchor (CWA) proteins recognized by a characteristic conserved peptide motif (LPXTG) and are mainly categorized as microbial surface components recognizing adhesive matrix molecules (MSCRAMMs). For example, serine–aspartate dipeptide repeat proteins SdrF, SdrG and SdrH, and the G5-E repeat protein family exemplified by Aap, mediate host–bacteria interactions (biotic attachment) [49,50]. The four genomes showed the presence of *sdrG* and *sdrH,* and notably AZ39 had a frameshifted *sdrG* (Table 5). Genomes of AZ22 and its close associates, *S. epidermidis* strains 1457 and ATCC 12228 in cluster 2, showed the presence of *sdrF* and *aap* genes. Strain 1457 is routinely used as a model organism for molecular studies of biofilms, whereas ATCC 12228 is a non-biofilm-forming strain [51,52]. *S. epidermidis* ST10002 harboured *aap* and the *icaABCDR* operon, which encodes PIA and is involved in the accumulation phase of biofilm formation. The *ica* operon is the hallmark of cluster 3, as it was present in all the genomes in this cluster (Table 5). This confirms our previous published data showing PIA production by this strain and not by AZ22, AZ39 or ST11003 [19]. Other proteins involved in cell–cell interactions include the elastin binding protein (Ebp) and the fibronectin binding protein (Ebh), a giant surface protein involved in biofilm accumulation [13]. Proteins encoded by the *icaABCDR* operon, Aap and the Ebh, are known to play pivotal roles in preventing *S. epidermidis* effector cell-mediated killing [53].

**Table 5.** Comparison of virulence factors amongst members of the three phylogenetic clusters

AZ39 and ST11003 cluster								
**Proteins (genes**)	**AZ39**	**14.1**.**R1**	**ST11003**	**SE95**				
Accessory gene regulator (*agrBDCA*)	*agrBC* (pseudo)	+	+	+				
Delta haemolysin (*hld*)	+	+	+	+				
Adhesin (*Aae*)	+	+	+	+				
Fibrinogen-binding adhesin SdrG C-terminal	Pseudo	−	−	−				
MSCRAMM family adhesin (*sdrG*)	−	+	+	−				
Fibrinogen-binding protein (*fbp*)	+	−	−	+				
MSCRAMM family adhesin (*sdrE*)	−	−	−	+				
MSCRAMM-like protein (*sdrH*)	−	+	+	+				
MSCRAMM family adhesin (*sdrC*)	−	−	−	−				
MSCRAMM family adhesin (*sdrF*)	−	−	−	−				
Elastin-binding protein (*ebp*)	+	+	+	+				
Hyperosmolarity resistance protein (*ebh*)	Frameshift	+	+	Frameshift				
Thermonuclease (*nuc*)	*nuc2*	*nuc1*, *nuc2*	*nuc1*, *nuc2*	*nuc2*				
*atlE*	+	+	+	+				
Staphylococcal enterotoxin type L	−	−	−	+				
Toxin	+			+				
Type VII secretion protein	+	+	+	+				
Accumulation-associated protein (*aap*)	−	−	−	−				
Intercellular adhesion protein (*icaABCDR*)	−	−	−	−				
Phenol soluble modulin (*psmα*)	+	−	−	+				
Phenol soluble modulin (*psmβ*)	4	5	5	6				
Phenol soluble modulin (*psmδ*)	−	+	+	+				
Phenol soluble modulin (*psmε*)	+	−	−	+				
Serine protease (*sspA*)	+	+	+	+				
Cysteine protease staphopain (*sspB*)	+	+	+	+				
dltABCD	+	+	+	+				
Multiple peptide resistance factor (*mprF*)	+	+	+	+				
ABC transporter (*vraGF*)	+	+	+	+				
Lipase (*gehCD*)	+	+	+	+				
**AZ22 cluster**								
	**AZ22**	**ATCC 12228**	**1457**	**CSF41498**	**GTH12**	**PM221**	**SEI**	
Accessory gene regulator (*agrBDCA*)	+	+	+	+	*agrBD* frameshift	*agrC* frameshift	+	
Delta haemolysin (*hld*)	+	+	+	+	+	+	+	
Adhesin (*Aae*)	+	+	+	+	+	+	+	
Fibrinogen-binding adhesin SdrG C-terminal	−	−	+	−	Frameshift	−	−	
MSCRAMM family adhesin (*sdrG*)	+	+	−	+	−	+	+	
Fibrinogen-binding protein (*fbp*)	−	−	−	−	−	−	−	
MSCRAMM family adhesin (*sdrE*)	−	−	−	−	−	−	−	
MSCRAMM-like protein (*sdrH*)	+	+	+	+	+	+	+	
MSCRAMM family adhesin (*sdrC*)	−	−	−	−	−	−	−	
MSCRAMM family adhesin (*sdrF*)	+	+	+	+	Pseudo	−	−	
Elastin-binding protein (*ebp*)	+	+	+	+	+	+	+	
Hyperosmolarity resistance protein (*ebh*)	+	Pseudo	Frameshift	+	Pseudo	Frameshift	+	
Thermonuclease (*nuc*)	*nuc2*	*nuc2*	*nuc2*	*nuc2*	*nuc2*	*nuc2*	*nuc2*	
Bifunctional autolysin *atlE*	+	+	+	+	+	+	+	
Staphylococcal enterotoxin type L	−	−	−	−	−	−	−	
Toxin	−	−	−	−	−	−	−	
Type VII secretion protein	−	−	−	−	−	−	−	
Accumulation-associated protein (*aap*)	+	+	+	−	Frameshift	−	−	
Intercellular adhesion protein (*icaABCDR*)	−	−	+	+	−	−	−	
Phenol soluble modulin (*psmα*)	−	−	−	−	−	−	−	
Phenol soluble modulin (*psmβ*)	7	7	6	7	6	5	4	
Phenol soluble modulin (*psmδ*)	+	+	+	+	+	+	+	
Phenol soluble modulin (*psmε*)	−	−	−	−	−	−	−	
Serine protease (*sspA*)	+	Frameshift	+	+	+	+	+	
Cysteine protease staphopain (*sspB*)	+	+	+	+	+	+	+	
dltABCD	+	+	+	+	+	+	+	
Multiple peptide resistance factor (*mprF*)	+	+	+	+	+	Frameshift	+	
ABC transporter (*vraGF*)	+	+	+	+	+	+	+	
Lipase (*gehCD*)	+	+	+	+	+	+	+	
**ST10002 cluster**								
	**O47**	**DAR1907**	**NCTC13 924**	**BPH0662**	**FDAARGOS_161**	**ST10002**	**RP62A**	**HD43**
Accessory gene regulator (*agrBDCA*)	Frameshift	+	+	+	+	+	+	*agrC* (pseudo)
Delta haemolysin (*hld*)	+	+	+	+	+	+	+	+
Adhesin (*Aae*)	+	+	+	+	+	+	+	+
Fibrinogen-binding adhesin SdrG C-terminal	−	+	+	−	−	−	−	−
MSCRAMM family adhesin (*sdrG*)	−	−	−	+	−	+	+	+
Fibrinogen-binding protein (*fbp*)	−	−	−	−	−	−	−	+
MSCRAMM family adhesin (*sdrE*)	−	−	−	−	−	−	−	−
SdrH family protein	+	−	−	−	−	−	+	+
MSCRAMM-like protein (*sdrH*)	−	+	+	+	+	+	Pseudo	−
MSCRAMM family adhesin (*sdrC*)	Pseudo	−	−	−	Frameshift	−	−	−
MSCRAMM family adhesin (*sdrF*)	+	+	−	+	+	−	−	−
Elastin-binding protein (*ebp*)	+	+	+	+	+	+	+	+
Hyperosmolarity resistance protein (*ebh*)	+	+	Frameshift	+	+	+	+	Pseudo
Thermonuclease (*nuc*)	*nuc2*	*nuc1,2,3*	*nuc1,2,3*	*nuc1,2*	*nuc2*	*nuc2*	*nuc1,2*	*nuc2*
Bifunctional autolysin *atlE*	+	+	+	+	+	+	+	+
Staphylococcal enterotoxin type L	−	−	−	−	−	−	−	−
Toxin	−	−	−	−	−	−	−	−
Type VII secretion protein	−	−	−	−	−	−	−	−
Accumulation-associated protein (*aap*)	Pseudo	−	−	−	+	+	+	−
Intercellular adhesion protein (*icaABCDR*)	+	+	+	+	+	+	+	+
Phenol soluble modulin (*psmα*)	−	−	−	−	−	−	−	−
Phenol soluble modulin (*psmβ*)	6	6	6	6	6	7	7	4
Phenol soluble modulin (*psmδ*)	+	+	+	+	+	+	+	+
Phenol soluble modulin (*psmε*)	−	−	−	−	−	−	−	−
Phenol-soluble modulin PSM-mec	−	+	+	+	−	−	+	−
Serine protease (*sspA*)	+	+	+	+	+	+	+	+
Cysteine protease staphopain (*sspB*)	+	+	+	+	+	+	+	+
dltABCD	+	+	+	+	+	+	+	+
Multiple peptide resistance factor (*mprF*)	+	+	+	+	+	+	+	+
ABC transporter (*vraGF*)	+	+	+	+	+	+	+	+
Lipase (*gehCD*)	+	+	+	+	*gehD* frameshift	+	+	+

*S. epidermidis* ST11003 and AZ39 formed a separate unique phylogenetic clade with *S. epidermidis* SE95 and 14.1 .R1 characterized by the presence of the type VII protein secretion system (T7SS) encoded by the *ess* locus (Table 5). The T7SS has been recently reported to play an important role in virulence in *S. aureus* [54,55]. 14.1 .R1 was isolated from the skin of a healthy person whereas SE95 was isolated from a child with bacteraemia [56,57]. Comparison of amino acid sequences of different factors involved in biofilm formation, such as SdrG, SdrH, Ebp, Ebh, AgrB, AgrC and Atl, amongst the four isolates showed significant differences (Table S3).

We have previously shown that the biofilm matrix of the three biofilm-positive strains, AZ22, AZ39 and ST10002, has eDNA, which is prone to degradation by thermonucleases. Our analysis showed that these three strains have the gene coding for thermonuclease Nuc2. Interestingly, the genome of strain ST11003 harbours genes coding for thermonucleases Nuc1 and Nuc2. Analysis of the presence of the *nuc1* gene in 24 fully assembled genomes showed that only eight *S*. *epidermidis* strains carry both *nuc1* and *nuc2* genes (Table 5, Fig. S3). Mutation in *nuc1* has been reported to lead to increased biofilm formation [58]. Furthermore, the *cidA*/*lrgAB* operons, which are involved in cell lysis and eDNA release, are present in all four genomes. Upstream from *lrgAB* are genes coding for the two-component signalling system, LytSR, which positively regulates transcription of the *lrgA* and *lrgB* genes [59].

*S. epidermidis* produces toxins known as phenol-soluble modulins (PSMs), including α-type peptides such as PSMα, PSMδ, PSMϵ, δ-toxin and PSM-mec, as well as β peptides PSMβ1 and PSMβ2. Amongst these virulence factors, α-type peptides have cytolytic activity; in particular, PSMδ is known to have strong cytolytic activity against leukocytes [60,61]. PSMδ was present in all *S. epidermidis* strains*,* including the 24 NCBI reference strains, except for AZ39. Beta peptides are involved in shaping biofilms by forming channels, and in the promotion of biofilm dispersal [62,63]. PSMβ was present in all the strains although its number varied from strain to strain (Table 5). Interestingly, PSMα and PSMε were only present in strain AZ39. Four ABC transporters for PSMs were observed in all four strains.

Recently, it has been reported that some *S. epidermidis* strains, such as SE95, are able to produce enterotoxins [57,64]. Interestingly, the AZ39 strain, which is closely related to *S. epidermidis* SE95, is the only one of the 24 genomes analysed in this study that harbours a toxin-encoding gene.

Furthermore, four YSIRK-type signal peptide-containing proteins and two YSIRK domain-containing triacylglycerol lipases, GehC and GehD, were observed in the genomes of *S. epidermidis* ST10002, AZ22 and AZ39. In *S. epidermidis* ST11003, three YSIRK-type signal peptide-containing proteins, two YSIRK signal domain/LPXTG anchor domain surface proteins, and the lipases GehC and GehD were present.

### Resistance against antibiotics and antimicrobial peptides

In *S. epidermidis* ST10002, antibiotic resistance genes identified in our analysis included genes that code for resistance to β-lactams (*blaZ*), macrolide–lincosamide–streptogramin B antibiotic (MLSB) [*msr*(A) and *mph*(C)], aminoglycosides [*aph*(3′)-III], fosfomycin (*fosB*) and streptothricin resistance determinant (SAT-4). The antiseptic resistance gene (*qacA*) was also identified in the ST10002 strain. In the genome of the ST11003 strain, we observed *fosB* and trimethoprim-sulfamethoxazole (*dfrG*) whereas only *fosB* is present in *S. epidermidis* AZ39 (Table 2). The *norA* gene, which represents the major facilitator superfamily (MFS) antibiotic efflux pump known for conferring resistance to fluoroquinolones, was present in all four strains.

One of the mechanisms used by *S. epidermidis* to evade the bactericidal actions of AMPs is the three-component AMP sensor system referred to as GraRSX [65]. GraRSX controls the expression of factors such as *dltABCD, mprF* and *vraFG,* which are involved in modification of the cell wall and change of charge of the cell membrane for repulsion of AMPs and excretion of AMP from the bacterial cells [66]. All four genomes showed the presence of *dltABCDX*, *mprF* and *vraFG* (Table 5).

## Discussion

*S. epidermidis* is one of the predominant bacteria of the skin microbiome and it is also an important member of the nasal microbiome [1,67]. This bacterium has an intriguing role in the survival and persistence of skin commensals by modulating the host immune system [68,69]. During blood collection, *S. epidermidis* can be introduced into blood from the skin of healthy blood donors resulting in contamination of blood components such as PCs. Transfusion of PCs to immunocompromised patients and people with haematological disorders has been vital in saving millions of lives worldwide. However, contamination of PCs with bacteria such as *S. epidermidis* poses a major safety risk to transfusion patients [3,5].

The PC storage environment offers a stressful environment for bacterial proliferation due to the bactericidal action of immune factors released by platelets [70]. We have demonstrated that *S. epidermidis* has adaptive mechanisms to resist the action of AMPs, plasma and killing by neutrophils [10,18, 71].

We have previously shown that all four *S. epidermidis* strains studied herein form protein-based biofilms when grown in PCs and that strain AZ39 was the strongest biofilm former [19,72]. Genomic analyses in this study unveiled the genetic determinants that could be involved in protein-based biofilm formation in PCs including Ebh, Ebp, SdrG, SdrH and Aap. For example, SdrG is a protein that binds fibrinogen, a plasma protein present in PCs, supporting our previous work which demonstrated that platelet storage containers preconditioned with plasma enhances bacterial attachment [73,74]. The *agrABCD* operon, which codes for proteins of the global negative regulator of biofilm formation, was found in its entirety in strains ST10002, ST11003 and AZ22; by contrast, the AZ39 strain harboured truncated *agrB* and *agrC*, probably explaining the strong biofilm formation capacity of the strain [75]. Similar findings were revealed by genomic analysis showing fragmented *agrC* in the strong biofilm former strain *S. epidermidis* O47 [45]. Another regulatory system, SaeRS, was only observed in ST11003. Deletion of this regulon in *S. epidermidis* 1457 increased its biofilm-forming ability [76] and we hypothesize that SaeRS may be repriming biofilm formation in ST11003 in media. As shown previously, biofilm-negative *S. epidermidis* strains, including ST11003, convert to a biofilm-positive phenotype in PCs [9,27] indicating regulation by other, as yet unknown, mechanisms.

Also, Loza-Correa *et al*. [19] reported eDNA to be one of the major components of biofilm matrices in ST10002, AZ22 and AZ39. The presence of eDNA in the matrix could be explained by the presence of *atl* and the *cidA/lrgAB* operon, which codes for holin/antiholin-like proteins involved in cell lysis and eDNA release during biofilm development [77,79].

Phylogenetic assignment provided insight into the identification of lineages and ecological niches. Strains ST10002 and AZ22 belong to clonal lineages ST16 and ST73. Strains from the ST16 clone have been reported to cause complicated infections along with strains from lineages ST2, ST5, ST7 and ST32 [22,80]. Based on WGS analyses, *S. epidermidis* isolates have been divided into two main phylogenetic clusters: A/C and B lineages [81]. A/C group isolates are known to be involved in colonization and infection and are more likely to cause nosocomial infections whereas group B members are mainly colonization isolates and commensals [82,83]. Isolates from the A/C group are adapted to colonize skin surfaces and grow under acidic and osmotic stress whereas B strains are adapted to survive in deeper skin layers and are resistant to bactericidal fatty acids [81]. Some genes have been linked to these two lineages; for example, the T7SS genes (*esaA, esaB, essA, essB, essC*) are reported to be present in B cluster strains [84]. Notably, ST11003 and AZ39 showed the presence of T7SS genes and thus they belong to the B cluster group. Also, T7SS genes are reported to produce an inhibitory toxin against the commensal skin bacterium *Cutibacterium acnes,* probably conferring a competitive advantage in the skin [85].

The arginine deiminase metabolism gene, *arcD*, encoding arginine/ornithine antiporter has been reported to be present in A/C cluster strains [81]. We observed two *arcD* copies in ST10002 and AZ22 and only one copy in ST11003 and AZ39, implying ST10002 and AZ22 belong to the A/C phylogenetic lineage. This extra copy, in addition to the ubiquitously present *arc* operon, has been reported to be important for *S. epidermidis* pH homeostasis and tolerance to low pH in *S. aureus* USA300 [86,87]. Another gene which has been linked to the A/C cluster is the hexose-6-phosphate:phosphate antiporter gene (*uhpT*) [88], which is present in strains ST10002 and AZ22 only. The biofilm (PIA-based) *icaADBC* operon has been associated with the A/C cluster and it was observed in *S. epidermidis* ST10002, which we have previously demonstrated to be PIA positive [19].

Overall, our WGS of ST10002, ST11003, AZ22 and AZ39 isolates has added to the growing repertoire of sequenced * S. epidermidis* strains, further broadening our understanding of the genetic basis of this species for virulence, resistance mechanisms against antibiotics and AMPs, and biofilm formation in PCs. Notably, the only biofilm-negative strain of the four studied herein, *S. epidermidis* ST11003, showed distinct genome features including the presence of genes coding for the SaeRS regulator system, two thermonucleases, and significant differences in amino acid similarity of virulence factors such as SdrG, SdrH and Agr with respect to the other three strains. How these differences are related to a negative biofilm formation phenotype requires further investigation. Notably, ST11003 along with AZ39 formed a separate unique lineage characterized by the presence of the T7SS. Importantly, all strains, independently of their genomic repertoire, are able to grow and form biofilms in PCs, indicating that multiple factors are probably involved to resist clearance by immune factors present in this harsh environment. Moreover, these ecologically flexible strains could be now used as model strains for studying biofilm formation and other physiological aspects of *S. epidermidis* under *in vitro* and *in vivo* studies in PCs or other environments.

## supplementary material

10.1099/acmi.0.000780.v3 Uncited Fig. S1.

10.1099/acmi.0.000780.v3 Uncited Table S1.

## Abbreviations

ACME: arginine catabolic mobile element
AMP: antimicrobial peptide
CRISPR: clustered regularly interspaced short palindromic repeat
eDNA: extracellular DNA
eDNA: extracellular DNA
MFS: major facilitator superfamily
MGE: mobile genetic element
MLSB: macrolide–lincosamide–streptogramin B antibiotic
MLST: multi-locus sequence typing
ncRNA: non-coding RNA
PC: platelet concentrate
PIA: polysaccharide intercellular adhesin
PSM: phenol-soluble modulin
RM: restriction modification
ST: sequence type
T7SS: type VII secretion system
WGS: whole genome sequencing

## References

[1] Byrd AL, Belkaid Y, Segre JA (2018). The human skin microbiome. Nat Rev Microbiol.

[2] National Nosocomial Infections Surveillance System (2004). National Nosocomial Infections Surveillance (NNIS) System Report, data summary from January 1992 through June 2004, issued October 2004. Am J Infect Control.

[3] Kou Y, Pagotto F, Hannach B, Ramirez-Arcos S (2015). Fatal false-negative transfusion infection involving a buffy coat platelet pool contaminated with biofilm-positive *Staphylococcus epidermidis*: a case report. Transfusion.

[4] Walther-Wenke G, Schrezenmeier H, Deitenbeck R, Geis G, Burkhart J (2010). Screening of platelet concentrates for bacterial contamination: spectrum of bacteria detected, proportion of transfused units, and clinical follow-up. Ann Hematol.

[5] Hong H, Xiao W, Lazarus HM, Good CE, Maitta RW (2016). Detection of septic transfusion reactions to platelet transfusions by active and passive surveillance. Blood.

[6] Ramirez-Arcos S, Goldman MC (2021). Bacterial contamination of platelet components. AABB Trans React.

[7] Jacobs MR, Good CE, Lazarus HM, Yomtovian RA (2008). Relationship between bacterial load, species virulence, and transfusion reaction with transfusion of bacterially contaminated platelets. Clin Infect Dis.

[8] Perez K, Patel R, Freitag NE (2018). Infection and Immunity.

[9] Greco C, Mastronardi C, Pagotto F, Mack D, Ramirez-Arcos S (2008). Assessment of biofilm-forming ability of coagulase-negative staphylococci isolated from contaminated platelet preparations in Canada. Transfusion.

[10] Greco C, Martincic I, Gusinjac A, Kalab M, Yang A-F (2007). *Staphylococcus epidermidis* forms biofilms under simulated platelet storage conditions. Transfusion.

[11] Rogers KL, Rupp ME, Fey PD (2008). The presence of icaADBC is detrimental to the colonization of human skin by *Staphylococcus epidermidis*. Appl Environ Microbiol.

[12] Rohde H, Burdelski C, Bartscht K, Hussain M, Buck F (2005). Induction of *Staphylococcus epidermidis* biofilm formation via proteolytic processing of the accumulation-associated protein by staphylococcal and host proteases. Mol Microbiol.

[13] Christner M, Franke GC, Schommer NN, Wendt U, Wegert K (2010). The giant extracellular matrix-binding protein of *Staphylococcus epidermidis* mediates biofilm accumulation and attachment to fibronectin. Mol Microbiol.

[14] Schommer NN, Christner M, Hentschke M, Ruckdeschel K, Aepfelbacher M, Bliska JB (2011). Infection and Immunity.

[15] Qin Z, Ou Y, Yang L, Zhu Y, Tolker-Nielsen T (2007). Role of autolysin-mediated DNA release in biofilm formation of *Staphylococcus epidermidis*. Microbiology.

[16] Stevens NT, Tharmabala M, Dillane T, Greene CM, O’Gara JP (2008). Biofilm and the role of the ICA operon and AAP in *Staphylococcus epidermidis* isolates causing neurosurgical meningitis. Clin Microbiol Infect.

[17] Hodgson SD, Greco-Stewart V, Jimenez CS, Sifri CD, Brassinga AKC (2014). Enhanced pathogenicity of biofilm-negative *Staphylococcus epidermidis* isolated from platelet preparations. Transfusion.

[18] Alabdullatif M, Atreya CD, Ramirez-Arcos S (2018). Antimicrobial peptides: an effective approach to prevent bacterial biofilm formation in platelet concentrates. Transfusion.

[19] Loza-Correa M, Ayala JA, Perelman I, Hubbard K, Kalab M, Das S (2019). PLoS One.

[20] Loza-Correa M, Yousuf B, Ramirez-Arcos S (2021). *Staphylococcus epidermidis* undergoes global changes in gene expression during biofilm maturation in platelet concentrates. Transfusion.

[21] Miragaia M, Thomas JC, Couto I, Enright MC, de Lencastre H (2007). Inferring a population structure for *Staphylococcus epidermidis* from multilocus sequence typing data. J Bacteriol.

[22] Lee JYH, Monk IR, Gonçalves da Silva A, Seemann T, Chua KYL (2018). Global spread of three multidrug-resistant lineages of *Staphylococcus epidermidis*. Nat Microbiol.

[23] Bouchami O, de Lencastre H, Miragaia M, Schaik van W (2016). PLoS One.

[24] Rolo J, Worning P, Nielsen JB, Bowden R, Bouchami O (2017). Evolutionary origin of the *Staphylococcal cassette* Chromosome MEC (SCC MEC). Antimicrob Agents Chemother.

[25] Onishi M, Urushibara N, Kawaguchiya M, Ghosh S, Shinagawa M (2013). Prevalence and genetic diversity of arginine catabolic mobile element (ACME) in clinical isolates of coagulase-negative staphylococci: identification of ACME type I variants in *Staphylococcus epidermidis*. Infect Genet Evol.

[26] O’Connor AM, McManus BA, Kinnevey PM, Brennan GI, Fleming TE (2018). Significant enrichment and diversity of the *Staphylococcal arginine* catabolic mobile element ACME in *Staphylococcus epidermidis* isolates from subgingival peri-implantitis sites and periodontal pockets. Front Microbiol.

[27] Taha M, Kohnen C, Mallya S, Kou Y, Zapata A (2018). Comparative characterisation of the biofilm-production abilities of *Staphylococcus epidermidis* isolated from human skin and platelet concentrates. J Med Microbiol.

[28] Chen S, Zhou Y, Chen Y, Gu J (2018). fastp: an ultra-fast all-in-one FASTQ preprocessor. Bioinformatics.

[29] Wick RR, Judd LM, Gorrie CL, Holt KE, Phillippy AM (2017). PLOS Computational Biology.

[30] Kolmogorov M, Yuan J, Lin Y, Pevzner PA (2019). Assembly of long, error-prone reads using repeat graphs. Nat Biotechnol.

[31] Wick RR, Holt KE (2019). Benchmarking of long-read assemblers for prokaryote whole genome sequencing. F1000Res.

[32] Ruan J, Li H (2020). Fast and accurate long-read assembly with wtdbg2. Nat Methods.

[33] Walker BJ, Abeel T, Shea T, Priest M, Abouelliel A (2014). Pilon: an integrated tool for comprehensive microbial variant detection and genome assembly improvement. PLoS One.

[34] Treangen TJ, Ondov BD, Koren S, Phillippy AM (2014). The Harvest suite for rapid core-genome alignment and visualization of thousands of intraspecific microbial genomes. Genome Biol.

[35] Tamura K, Stecher G, Peterson D, Filipski A, Kumar S (2013). MEGA6: Molecular Evolutionary Genetics Analysis version 6.0. Mol Biol Evol.

[36] Grissa I, Vergnaud G, Pourcel C (2007). CRISPRFinder: a web tool to identify clustered regularly interspaced short palindromic repeats. Nucleic Acids Res.

[37] Alcock BP, Raphenya AR, Lau TTY, Tsang KK, Bouchard M (2020). CARD 2020: antibiotic resistome surveillance with the comprehensive antibiotic resistance database. Nucleic Acids Res.

[38] Arndt D, Grant JR, Marcu A, Sajed T, Pon A (2016). PHASTER: a better, faster version of the PHAST phage search tool. Nucleic Acids Res.

[39] Chen L, Zheng D, Liu B, Yang J, Jin Q (2016). VFDB 2016: hierarchical and refined dataset for big data analysis--10 years on. Nucleic Acids Res.

[40] Herbig A, Nieselt K (2011). nocoRNAc: characterization of non-coding RNAs in prokaryotes. BMC Bioinformatics.

[41] Tatusova T, DiCuccio M, Badretdin A, Chetvernin V, Nawrocki EP (2016). NCBI prokaryotic genome annotation pipeline. Nucleic Acids Res.

[42] Jolley KA, Bray JE, Maiden MCJ (2018). Open-access bacterial population genomics: BIGSdb software, the PubMLST.org website and their applications. Wellcome Open Res.

[43] Thomas JC, Vargas MR, Miragaia M, Peacock SJ, Archer GL (2007). Improved multilocus sequence typing scheme for *Staphylococcus epidermidis*. J Clin Microbiol.

[44] Rosenstein R, Götz F (2012). Between Pathogenicity and Commensalism.

[45] Raue S, Fan S-H, Rosenstein R, Zabel S, Luqman A (2020). The genome of *Staphylococcus epidermidis* O47. Front Microbiol.

[46] Schoenfelder SMK, Lange C, Prakash SA, Marincola G, Lerch MF (2019). The small non-coding RNA RsaE influences extracellular matrix composition in *Staphylococcus epidermidis* biofilm communities. PLoS Pathog.

[47] Marraffini LA, Sontheimer EJ (2008). CRISPR interference limits horizontal gene transfer in staphylococci by targeting DNA. Science.

[48] Cave R, Misra R, Chen J, Wang S, Mkrtchyan HV (2021). Comparative genomics analysis demonstrated a link between *Staphylococci* isolated from different sources: a possible public health risk. Front Microbiol.

[49] Büttner H, Mack D, Rohde H (2015). Structural basis of *Staphylococcus epidermidis* biofilm formation: mechanisms and molecular interactions. Front Cell Infect Microbiol.

[50] Foster TJ (2020). Surface proteins of *Staphylococcus epidermidis*. Front Microbiol.

[51] Zhang Y-Q, Ren S-X, Li H-L, Wang Y-X, Fu G (2003). Genome-based analysis of virulence genes in a non-biofilm-forming *Staphylococcus epidermidis* strain (ATCC 12228). Mol Microbiol.

[52] Galac MR, Stam J, Maybank R, Hinkle M, Mack D (2017). Complete genome sequence of *Staphylococcus epidermidis* 1457. Genome Announc.

[53] Le KY, Park MD, Otto M (2018). Immune evasion mechanisms of *Staphylococcus epidermidis* biofilm infection. Front Microbiol.

[54] Cao Z, Casabona MG, Kneuper H, Chalmers JD, Palmer T (2016). The type VII secretion system of *Staphylococcus aureus* secretes a nuclease toxin that targets competitor bacteria. Nat Microbiol.

[55] Bowman L, Palmer T (2021). The type VII secretion system of *Staphylococcus*. Annu Rev Microbiol.

[56] Lassen SB, Lomholt HB, Brüggemann H (2017). Complete genome sequence of a *Staphylococcus epidermidis* strain with exceptional antimicrobial activity. Genome Announc.

[57] Argemi X, Nanoukon C, Affolabi D, Keller D, Hansmann Y (2018). Comparative genomics and identification of an enterotoxin-bearing pathogenicity Island, SEPI-1/SECI-1, in *Staphylococcus epidermidis* pathogenic strains. Toxins.

[58] Schilcher K, Horswill AR (2020). Staphylococcal biofilm development: structure, regulation, and treatment strategies. Microbiol Mol Biol Rev.

[59] Brunskill EW, Bayles KW (1996). Identification and molecular characterization of a putative regulatory locus that affects autolysis in *Staphylococcus aureus*. J Bacteriol.

[60] Cheung GYC, Rigby K, Wang R, Queck SY, Braughton KR (2010). *Staphylococcus epidermidis* strategies to avoid killing by human neutrophils. PLoS Pathog.

[61] Cheung GYC, Joo H-S, Chatterjee SS, Otto M (2014). Phenol-soluble modulins--critical determinants of staphylococcal virulence. FEMS Microbiol Rev.

[62] Otto M (2014). Phenol-soluble modulins. Int J Med Microbiol.

[63] Le KY, Villaruz AE, Zheng Y, He L, Fisher EL (2019). Role of phenol-soluble modulins in *Staphylococcus epidermidis* biofilm formation and infection of indwelling medical devices. J Mol Biol.

[64] Nanoukon C, Affolabi D, Keller D, Tollo R, Riegel P (2018). Characterization of human type C enterotoxin produced by clinical *S. epidermidis* isolates. Toxins (Basel).

[65] Li M, Lai Y, Villaruz AE, Cha DJ, Sturdevant DE (2007). Gram-positive three-component antimicrobial peptide-sensing system. Proc Natl Acad Sci U S A.

[66] Joo HS, Otto M (2015). Mechanisms of resistance to antimicrobial peptides in staphylococci. Biochim Biophys Acta.

[67] Liu Q, Liu Q, Meng H, Lv H, Liu Y (2020). *Staphylococcus epidermidis* contributes to healthy maturation of the nasal microbiome by stimulating antimicrobial peptide production. Cell Host Microbe.

[68] PrabhuDas M, Adkins B, Gans H, King C, Levy O (2011). Challenges in infant immunity: implications for responses to infection and vaccines. Nat Immunol.

[69] Naik S, Bouladoux N, Linehan JL, Han S-J, Harrison OJ (2015). Commensal-dendritic-cell interaction specifies a unique protective skin immune signature. Nature.

[70] Fitzgerald JR, Foster TJ, Cox D (2006). The interaction of bacterial pathogens with platelets. Nat Rev Microbiol.

[71] Taha M, Kyluik-Price D, Kumaran D, Scott MD, Toyofuku W (2019). Bacterial survival in whole blood depends on plasma sensitivity and resistance to neutrophil killing. Transfusion.

[72] Stepanović S, Vuković D, Hola V, Di Bonaventura G, Djukić S (2007). Quantification of biofilm in microtiter plates: overview of testing conditions and practical recommendations for assessment of biofilm production by staphylococci. APMIS.

[73] Loza-Correa M, Kalab M, Yi Q-L, Eltringham-Smith LJ, Sheffield WP (2017). Comparison of bacterial attachment to platelet bags with and without preconditioning with plasma. Vox Sang.

[74] Yousuf B, Pasha R, Pineault N, Ramirez-Arcos S (2022). Contamination of platelet concentrates with *Staphylococcus aureus* induces significant modulations in platelet functionality. Vox Sang.

[75] Grande R, Nistico L, Sambanthamoorthy K, Longwell M, Iannitelli A (2014). Temporal expression of agrB, cidA, and alsS in the early development of *Staphylococcus aureus* UAMS-1 biofilm formation and the structural role of extracellular DNA and carbohydrates. Pathog Dis.

[76] Lou Q, Zhu T, Hu J, Ben H, Yang J (2011). Role of the SaeRS two-component regulatory system in *Staphylococcus epidermidis* autolysis and biofilm formation. BMC Microbiol.

[77] Bayles KW (2007). The biological role of death and lysis in biofilm development. Nat Rev Microbiol.

[78] Rice KC, Mann EE, Endres JL, Weiss EC, Cassat JE (2007). The cidA murein hydrolase regulator contributes to DNA release and biofilm development in *Staphylococcus aureus*. Proc Natl Acad Sci U S A.

[79] Sadykov MR, Bayles KW (2012). The control of death and lysis in staphylococcal biofilms: a coordination of physiological signals. Curr Opin Microbiol.

[80] Shelburne SA, Dib RW, Endres BT, Reitzel R, Li X (2020). Whole-genome sequencing of *Staphylococcus epidermidis* bloodstream isolates from a prospective clinical trial reveals that complicated bacteraemia is caused by a limited number of closely related sequence types. Clin Microbiol Infect.

[81] Espadinha D, Sobral RG, Mendes CI, Méric G, Sheppard SK (2019). Distinct phenotypic and genomic signatures underlie contrasting pathogenic potential of *Staphylococcus epidermidis* clonal lineages. Front Microbiol.

[82] Conlan S, Mijares LA, Becker J, Blakesley RW, Bouffard GG (2012). *Staphylococcus epidermidis* pan-genome sequence analysis reveals diversity of skin commensal and hospital infection-associated isolates. Genome Biol.

[83] Méric G, Miragaia M, de Been M, Yahara K, Pascoe B (2015). Ecological overlap and horizontal gene transfer in *Staphylococcus aureus* and *Staphylococcus epidermidis*. Genome Biol Evol.

[84] Lai Y, Villaruz AE, Li M, Cha DJ, Sturdevant DE (2007). The human anionic antimicrobial peptide dermcidin induces proteolytic defence mechanisms in staphylococci. Mol Microbiol.

[85] Christensen GJM, Scholz CFP, Enghild J, Rohde H, Kilian M (2016). Antagonism between *Staphylococcus epidermidis* and propionibacterium acnes and its genomic basis. BMC Genomics.

[86] Thurlow LR, Joshi GS, Clark JR, Spontak JS, Neely CJ (2013). Functional modularity of the arginine catabolic mobile element contributes to the success of USA300 methicillin-resistant *Staphylococcus aureus*. Cell Host Microbe.

[87] Lindgren JK, Thomas VC, Olson ME, Chaudhari SS, Nuxoll AS (2014). Arginine deiminase in *Staphylococcus epidermidis* functions to augment biofilm maturation through pH homeostasis. J Bacteriol.

[88] Park JY, Kim JW, Moon BY, Lee J, Fortin YJ (2015). Characterization of a novel two-component regulatory system, HptRS, the regulator for the hexose phosphate transport system in *Staphylococcus aureus*. Infect Immun.

