# Supplementary material for: Comparative virulome analysis of four Staphylococcus epidermidis strains from human skin and platelet concentrates using whole genome sequencing
Source: Access Microbiol. 2024 Apr 3;6(4):000780.v3. doi: 10.1099/acmi.0.000780.v3 (PMC11083402; doi:10.1099/acmi.0.000780.v3)
Supplement: Uncited Fig. S1. [file acmi-6-00780-s001.pdf]

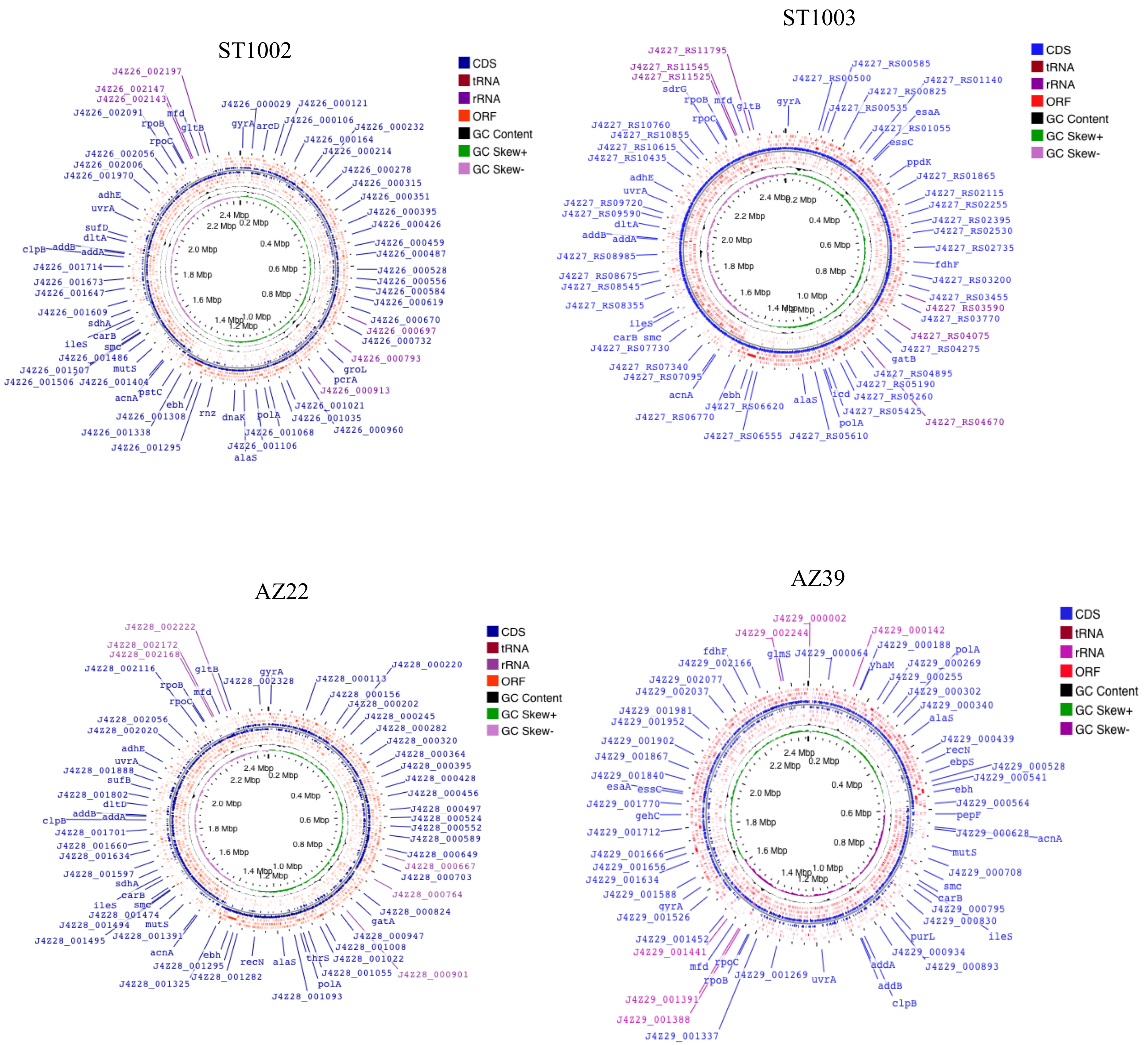

Supplementary Figure 1. Circular genome plots of four *S. epidermidis* strains. The plot was generated using CGView.

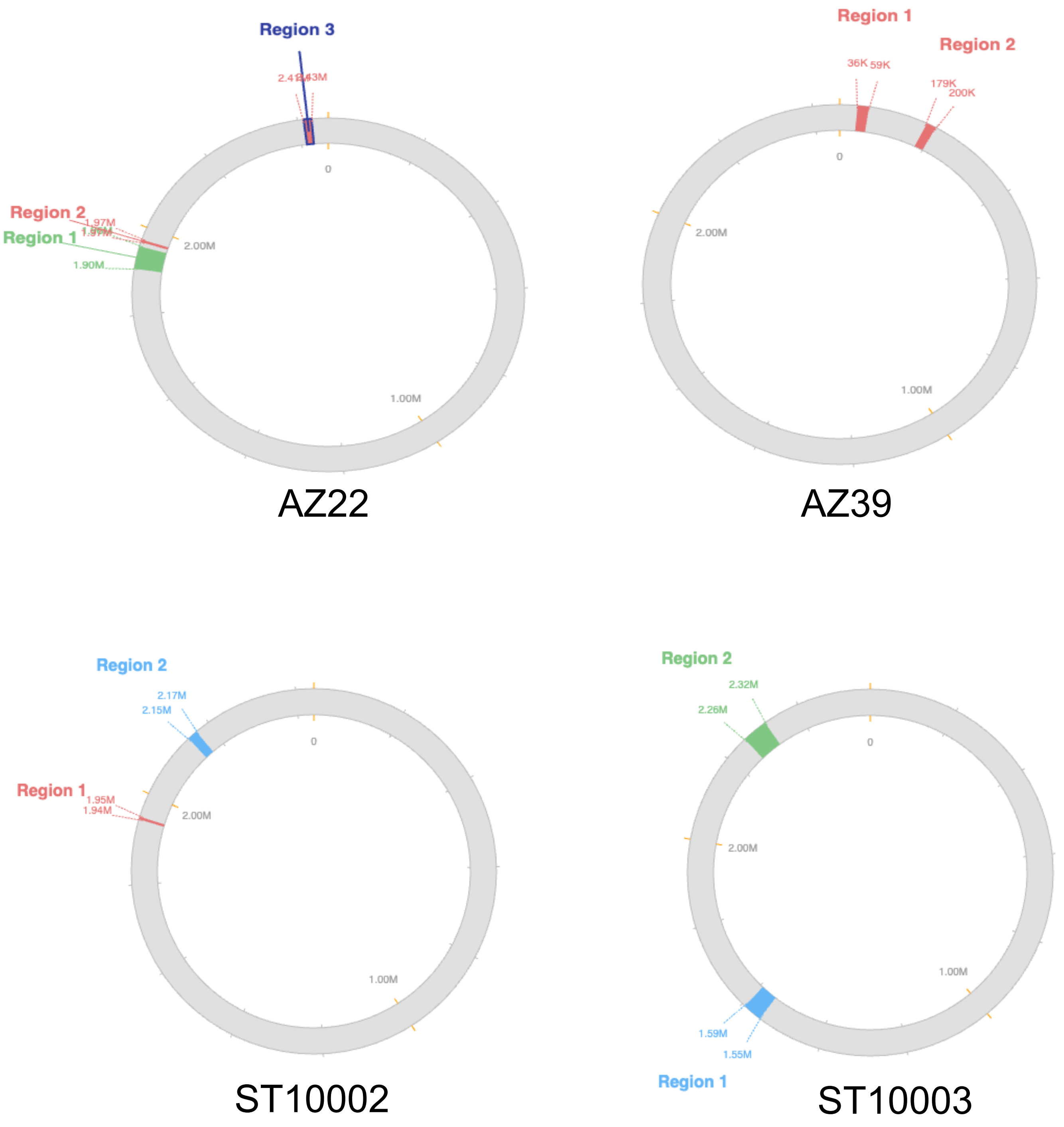

Supplementary Figure 2. Number of Prophages regions and their sizes in the genomes of *S. epidermidis* AZ22, AZ39, ST10002, and ST10003 strains isolated from skin and platelet concentrates.

```
RP62A           --MKVGEHLYTYKAKCTKVIDGDTLDIVIDFGFNTYGKRRVRLLGVDTPERSQTNYKEAT   58
NCTC13924       --MKVGEHLYTYKAKCTKVIDGDTLDIVIDFGFNTYGKRRVRLLGVDTPERSQTNYKEAT   58
BPH0662         --MKVGEHLYTYKAKCTKVIDGDTLDIVIDFGFNTYGKRRVRLLGVDTPERSQTNYKEAT   58
DAR1907         --MKVGEHLYTYKAKCTKVIDGDTLDIVIDFGFNTYGKRRVRLLGVDTPERSQTNYKEAT   58
FDARGOS_529     ---MNINNLYTYKATCTNVVDGDTLDILLDCGFDTYAKRRVRLLGVDTPERGQDKFKEAT   57
HD66            ---MNINNLYTYKATCTNVVDGDTLDILLDCGFDTYAKRRVRLLGVDTPERGQENYKEAT   57
ST11003         ---MNINNLYTYKATCTNVVDGDTLDILLDCGFDTYAKRRVRLLGVDTPERGQENYKEAT   57
14.1.R1         MFTIDINNLYTYKATCTNVVDGDTLDILLDCGFDTYAKRRVRLLGVDTPERGQENYKEAT   60
HD33            MFTIDINNLYTYKATCTNVVDGDTLDILLDCGFDTYAKRRVRLLGVDTPERGQENYKEAT   60
                      ::******.**:*:*******::* **:**.**************.* ::****

RP62A           QFTRSCVENKDIYIQSHKSDSFGRYLAKVWFEN-ERCLNEELKKSGLLKENSKWNKEE     115
NCTC13924       QFTRSCVENKDIYIQSHKSDSFGRYLAKVWFEN-ERCLNEELKKSGLLKENSKWNKEE     115
BPH0662         QFTRSCVENKDIYIQSHKSDSFGRYLAKVWFEN-ERCLNEELKKSGLLKENSKWNKEE     115
DAR1907         QFTRSCVENKDIYIQSHKSDSFGRYLAKVWFEN-ERCLNEELKKSGLLKENSKWNKEE     115
FDARGOS_529     AFTRECVEHKDIYVQTYKSDVFGRYLANVWYEDGRRNLNDDLRDAGLLKENSKWNEG-     114
HD66            ALTRSCVENKDIYVQTYKSDVFGRYLANVWYENGQRSLNDDLRSAGLLKENSKWNEG-     114
ST11003         ALTRSCVENKDIYVQTYKSDVFGRYLANVWYEDGKYCLNDELRNAGLLKPKSKWNEDE     115
14.1.R1         ALTRSCVENKDIYVQTYKSDVFGRYLANVWYEDGKRSLNDELRNAGLLKPKSKWNEG-     117
HD33            VLTRSCVENKDIYVQTYKSDVFGRYLANVWYEDGKYCLNDELRKAGLLKENSKWNEG-     117
                 :**.***:****:*::*** ******:**:*: .  **::*:.:**** :****:
```

1: RP62A	100.00	100.00	100.00	100.00	68.14	71.68	70.18	69.30	71.93
2: NCTC13924	100.00	100.00	100.00	100.00	68.14	71.68	70.18	69.30	71.93
3: BPH0662	100.00	100.00	100.00	100.00	68.14	71.68	70.18	69.30	71.93
4: DAR1907	100.00	100.00	100.00	100.00	68.14	71.68	70.18	69.30	71.93
5: FDARGOS_529	68.14	68.14	68.14	68.14	100.00	91.23	87.72	87.72	87.72
6: HD66	71.68	71.68	71.68	71.68	91.23	100.00	92.11	92.98	92.11
7: ST11003	70.18	70.18	70.18	70.18	87.72	92.11	100.00	95.61	93.86
8: 14.1.R1	69.30	69.30	69.30	69.30	87.72	92.98	95.61	100.00	94.87
9: HD33	71.93	71.93	71.93	71.93	87.72	92.11	93.86	94.87	100.00

Supplementary Figure 3. Thermonuclease 1 (Nuc1) was present in only 8 genomes from 24 assembled genomes available in NCBI. Percent amino acid similarity was determined using Clustal Omega.
