# Supplementary material for: Comparative virulome analysis of four Staphylococcus epidermidis strains from human skin and platelet concentrates using whole genome sequencing
Source: Access Microbiol. 2024 Apr 3;6(4):000780.v3. doi: 10.1099/acmi.0.000780.v3 (PMC11083402; doi:10.1099/acmi.0.000780.v3)
Supplement: Uncited Table S1. [file acmi-6-00780-s002.pdf]

Supplementary Table 1. Sequence type of four isolates *S. epidermidis* ST-11003, AZ39, AZ22 and ST-10002 along with 24 reference genomes.

Isolate	ST	Source	Country	Year
S. epidermidis SE95	NA	Blood	France	2014
S. epidermidis ST-11003	1174	Blood	Canada	2011
S. epidermidis 14.1.R1	1018	Skin	Denmark	2015
S. epidermidis AZ39	1175	Skin	Canada	2015
S. epidermidis ATCC_14990	NA	Skin	USA	2019
S. epidermidis NBRC 100911	5	NA	Japan	2019
S. epidermidis 949_S8	NA	NA	South Africa	2012
S. epidermidis HD66	87	Skin	Germany	2018
S. epidermidis HD33	87	Skin	Germany	2018
S. epidermidis FDAARGOS_153	5	Skin	USA	2014
S. epidermidis PM221	184	NA	Finland	2014
S. epidermidis SEI	1016	NA	USA	2010
S. epidermidis ATCC 12228	8	NA	China	2003
S. epidermidis ATCC 12228	8	NA	South Korea	2019
S. epidermidis 1457	NA	NA	Germany	1990
S. epidermidis AZ22	73	Skin	Canada	2015
S. epidermidis CSF41498	297	Cerebrospinal fluid	Ireland	2000
S. epidermidis GTH12	769	Skin	Brazil	2017
S. epidermidis O47	2	Orthopedic device	Germany	2011
S. epidermidis DAR1907	2	Skin	USA	2007
S. epidermidis NCTC13924	2	Blood	UK	2013
S. epidermidis BPH0662	2	Skin	Australia	2016
S. epidermidis FDAARGOS_161	20	Skin	USA	2014
S. epidermidis ST-10002	16	Blood	Canada	2010
S. epidermidis RP62A	10	Catheter	USA	2004
S. epidermidis HD43	23	Skin	Germany	2018
S. epidermidis CDC120	20	Skin	South Korea	2017
S. epidermidis CDC121	20	Skin	South Korea	2017

NA- Not Available
